# Decoronation followed by dental implants placement: fundamentals, applications and explanations

**DOI:** 10.1590/2177-6709.23.1.024-036.oin

**Published:** 2018

**Authors:** Alberto Consolaro, Paulo Domingos Ribeiro, Maurício A. Cardoso, Dario A. Oliveira Miranda, Monica Salfatis

**Affiliations:** 1 Universidade de São Paulo, Faculdade de Odontologia de Bauru (Bauru/SP, Brazil).; 2 Universidade de São Paulo, Faculdade de Odontologia de Ribeirão Preto, Programa de Pós-graduação em Odontopediatria (Ribeirão Preto/SP, Brazil).; 3 Universidade Sagrado Coração, Programa de Pós-graduação em Biologia, Division of Oral and Maxillofacial Surgery (Bauru/SP, Brazil).; 4 Faculdade de Medicina e Odontologia São Leopoldo Mandic, Programa de Pós-graduação em Ortodontia (Campinas/SP, Brazil).; 5 Universidade Estadual de Feira de Santana, Curso de Odontologia (Feira de Santana/BA, Brazil).; 6 Private practice (São Paulo/SP, Brazil).

**Keywords:** Decoronation, Coronectomy, Dental implants, Dentoalveolar ankylosis, Replacement root resorption

## Abstract

Dental arches areas with teeth presenting dentoalveolar ankylosis and replacement root resorption can be considered as presenting normal bone, in full physiological remodeling process; and osseointegrated implants can be successfully placed. Bone remodeling will promote osseointegration, regardless of presenting ankylosis and/or replacement root resorption. After 1 to 10 years, all dental tissues will have been replaced by bone. The site, angulation and ideal positioning in the space to place the implant should be dictated exclusively by the clinical convenience, associated with previous planning. One of the advantages of decoronation followed by dental implants placement in ankylosed teeth with replacement resorption is the maintenance of bone volume in the region, both vertical and horizontal. If possible, the buccal part of the root, even if thin, should be preserved in the preparation of the cavity for the implant, as this will maintain gingival tissues looking fully normal for long periods. In the selection of cases for decoronation, the absence of microbial contamination in the region - represented by chronic periapical lesions, presence of fistula, old unconsolidated root fractures and active advanced periodontal disease - is important. Such situations are contraindications to decoronation. However, the occurrence of dentoalveolar ankylosis and replacement resorption without contamination should neither change the planning for implant installation, nor the criteria for choosing the type and brand of dental implant to be used. Failure to decoronate and use dental implants has never been reported.

## RESUMO

Áreas dos maxilares com dentes em anquilose alveolodentária e reabsorção dentária por substituição podem ser consideradas como portadoras de osso normal, em pleno processo fisiológico contínuo de remodelação; e os implantes osseointegráveis podem ser aplicados com sucesso. A remodelação óssea promoverá sua osseointegração, independentemente de haver raízes em anquilose e/ou em reabsorção por substituição. Após 1 a 10 anos, todos os tecidos dentários terão sido substituídos por osso. O local, a angulação e o posicionamento ideal no espaço para se colocar o implante devem ser ditados pela conveniência clínica associada, exclusivamente, ao planejamento prévio. Uma das vantagens da decoronação com colocação imediata de implantes em dentes anquilosados e com reabsorção por substituição é a manutenção do volume ósseo na região, tanto vertical quanto horizontalmente. Se possível, deve-se preservar, na preparação da cavidade para o implante, a parte vestibular da raiz, mesmo que fina; isso deixará os tecidos gengivais com aspecto de plena normalidade por longos períodos. O importante na seleção de casos para a decoronação é a ausência de contaminação microbiana na região, representada por lesões periapicais crônicas, presença de fístula, fraturas radiculares antigas não consolidadas e doença periodontal avançada ativa. Essas situações são contraindicações para a decoronação. A ocorrência de anquilose alveolodentária e reabsorção por substituição sem contaminação não deve mudar o planejamento para instalação de implantes, nem mesmo os critérios de escolha do tipo e marca de implante dentário a ser utilizado. Nunca foi relatado fracasso na decoronação e uso de implantes dentários.

The extraction of teeth with ankylosis and replacement root resorption promotes greater post-surgical alveolar bone loss than the extraction of teeth with preserved periodontal ligament. This greater bone loss makes it difficult to reestablish the aesthetics and function of the region, with sequelae due to loss of vertical and/or horizontal volume of the alveolar process.

Correction of sequelae after the extraction of teeth with ankylosis and replacement resorption usually requires soft and hard tissues grafts and, in these cases, implies longer surgical procedures and greater material cost.^3^ ^,^ ^23^ Decoronation was developed as an alternative treatment to the extraction of ankylosed teeth, in order to overcome these undesirable effects. Decoronation was introduced in the literature by Malmgren et al,^13^ in 1984 and represents the coronectomy of ankylosed teeth 2 mm below the cementoenamel junction. The affected tooth will tend to disappear in 1 to 10 years after the procedure, with or without implants.^17^

In the initial concept of decoronation, the second phase, represented by the late or immediate placement of dental implants, was not present - a natural evolution of the procedure and knowledge. Decoronation is poorly explored academically, and most clinicians do not know about this possibility, with or without the placement of osseointegrated implants.^17^

With the immediate application of the implants after decoronation, the bone volume and soft tissue maintenance, vertically and horizontally, is further favored, benefiting the proper restoration of aesthetics and function. The same occurs when implants are applied later.^8^ ^,^ ^9^ ^,^ ^11^ ^,^ ^14^ ^,^ ^22^ This favoring already happens when just the decoronation is done, without the placement of implants;^4^ ^,^ ^10^ ^,^ ^12^ ^,^ ^20^ ^,^ ^21^ but with the implants, this seems more prominent.

In the present article, we have simply sought to provide a basis for understanding why dentoalveolar ankylosis and its consequent replacement root resorption should be regarded as natural, despite the inconvenience of consuming the roots and replacing them with bone.^7^ It is also the purpose of this article to explain biologically - from inferences, unfoldings, extrapolations and interrelations - why, even with dentoalveolar ankylosis and replacement root resorption, implants can be placed immediately after decoronation, even when in the middle of the bone-tooth replacement process, without disturbing it and, at the same time, causing osseointegration to occur normally.

## Why should dentoalveolar ankylosis and replacement root resorption be considered natural and almost “normal”?

The search for understanding how species evolved biologically is part of a science known as Phylogeny, or Phylogenesis. The evolution of the mouth and the appearance of teeth are studied by this science.^16^ In the evolution of the species, the mouths of the very inferior animals were covered by epithelial coatings, but the needed food was hard, and these coatings started to thicken, especially over the parts of bony ridges where they chewed, almost creating focal callosities. From this need for harder foods, the underlying bone needed to thicken, condense and rise in processes or projections to help cutting, grinding and tearing up what one had to eat. In the evolution of the species, these bone processes and projections now no longer needed to be covered by mucous membranes and other types of coatings. The hard, dense part of the bone would need to be exposed in the mouth and act directly on the food. To make them more efficient, the evolution of the species promoted the appearance of enamel on these teeth: now they were very hard and perfect to act on food.

***And so periodontal ligament arose*** . The ‘bone teeth’ exposed in the mouth were stiff and broke, which probably hurt a lot. In evolution, between this bone - dense and different (to chew) - and the underlying bone, membranes and bands of connective tissue appeared to cushion the impacts as rudimentary periodontal ligaments. The epithelial lining went very early on to give rise to the dental lamina in the form of buds or germs, from which the teeth would originate as they are today.

**Figure 1:**
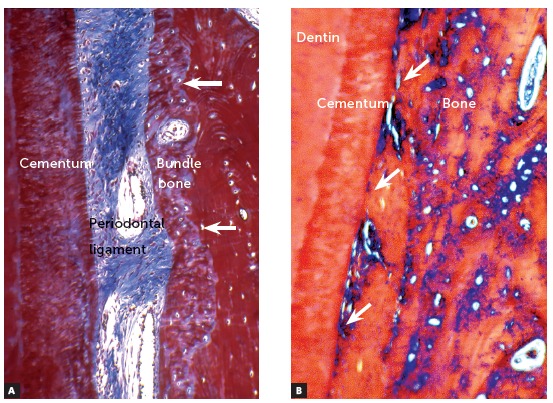
In A, tooth supporting structures are noted in the dental alveolus, with organization maintained by the epithelial rests of Malassez that release EGF, keeping the bundle bone (arrows) and cement apart, avoiding dentoalveolar ankylosis with subsequent replacement root resorption. In B, there is dentoalveolar ankylosis, with bone being formed and interacting with the root surface (arrows), still without resorptive process (HE and TM = 40X).

The tooth was responsible for creating its structures of interaction with the neighboring bone. The outermost part of the dental germ now gives rise to the cementum, periodontal ligament, and the coating, or “plaster”, of the alveolus - called bundle bone or alveolar bone itself (Fig 1).

***Bones and teeth have the same phylogenetic origins: they are related tissues*** . The normal situation would be that cementum, periodontal ligament and bundle bone fraternize at any moment, messing the periodontal organization as we know it. In order to avoid the occurrence of dentoalveolar ankylosis and consequent replacement root resorption, phylogenetic evolution distributed epithelial strands in the form of a basketball net through the periodontal ligament. These strands are the epithelial rests of Malassez.

The epithelia continuously release a substance that induces their cells to proliferate at all times, so that they are renewed in the processes of desquamation of the surfaces. This substance is called EGF, or epidermal growth factor, and induces epithelial cell proliferation; but if it finds bone cells, it stimulates resorption by the clasts.^7^

In the EGF-soaked periodontal ligament, the bundle bone is formed in layers towards the tooth; but the cement will never be touched, because these layers will almost immediately be reabsorbed by the clasts. Cement and bundle bone are doomed to never touch (Fig 1) and the factor for this distancing is the EGF produced by the epithelial rests of Malassez.

**Figure 2:**
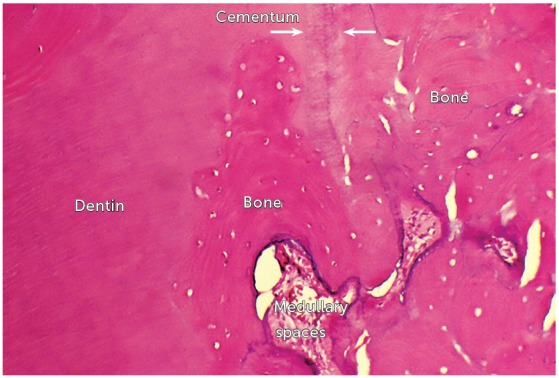
Replacement root resorption subsequent to dentoalveolar ankylosis, with bone forming and merging with the cementum (arrows) and dentin, replacing them (HE = 40X).

**Figure 3:**
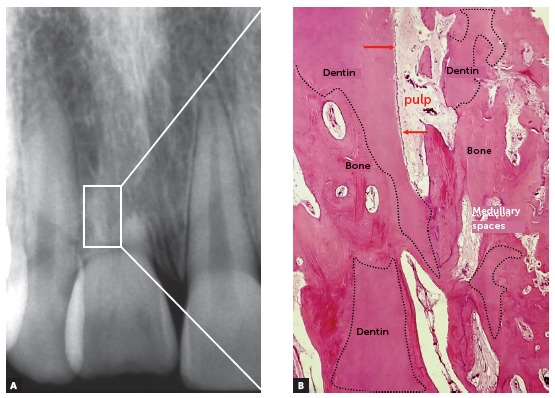
In replacement root resorption, dental fragments remain in place for long periods as they are reabsorbed in the context of bone remodeling. In some dentin fragments (dashed lines), areas with odontoblastic layer (arrows) and pulp are still observed. In the areas already substituted, bone with its trabeculae and medullary spaces is seen with normal organization and structures (HE, B = 20X).

**Figure 4:**
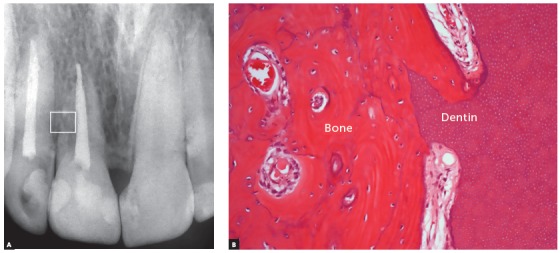
Replacement resorption in tooth with late dental reimplantation in a 17-year-old patient. It should be noted that the dental structure was not contaminated and replacement root resorption occurred slowly, without the tooth losing support in full function. The cervical bone level has been preserved and the bone tissue related to the replacement of the root dental tissues is normal in the imaging exams. Microscopically, the tissues are normal in their organization and structure, without inflammation (HE, 40X).

**Figure 5:**
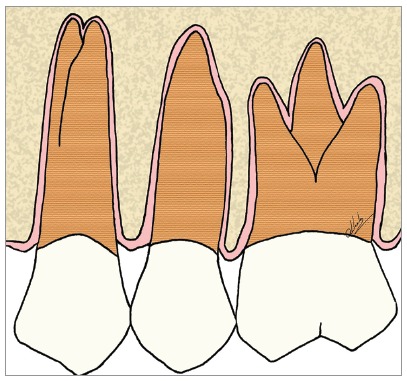
Scheme of the periodontal and bony structures, emphasizing the periodontal space, in pink.

**Figure 6:**
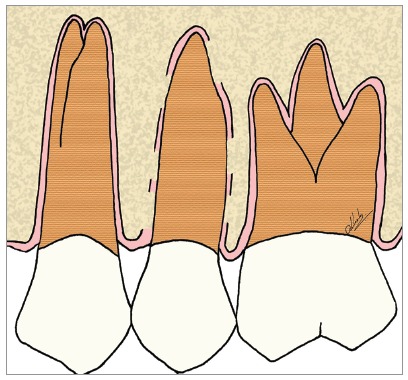
Scheme of focal areas of dentoalveolar ankylosis in the maxillary second premolar.

**Figure 7:**
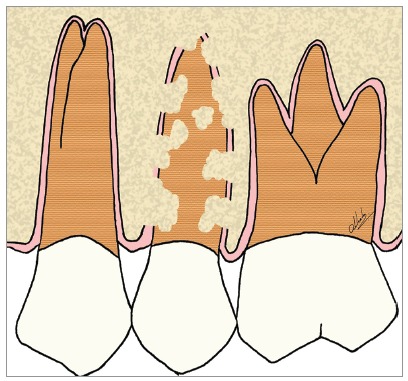
Scheme of focal areas with replacement resorption, in the maxillary second premolar.

**Figure 8:**
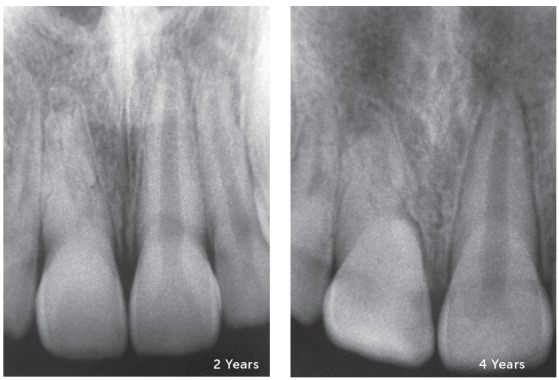
Replacement resorption in the maxillary central incisor, with preservation of vitality, as it can occur in dental trauma of lesser intensity of the concussion type. Radiographs were performed as control, after 2 and 4 years. The bone merges with the root structure, gradually replacing it.

**Figure 9:**
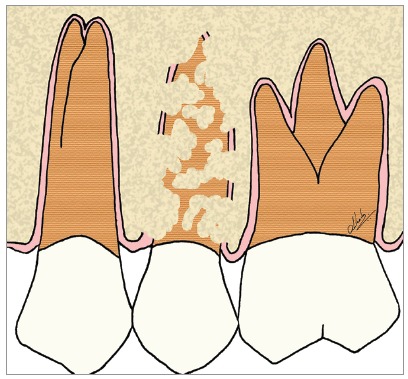
Scheme of advanced replacement resorption in the maxillary second premolar.

***Dentoalveolar ankylosis and replacement root resorption arise*** . The elimination of the epithelial rests of Malassez occurs only by dental traumatism. Necrosis of the epithelial rests of Malassez occurs at isolated sites of the periodontal ligament; sometimes in larger segments. It is at these points that the bone gets to the root surface (Fig 2 to 9). Radiographs may reveal the presence of ankylosis only if there is at least 20% of affected root surface contacting with bone^1^ (Figs 3, 4, 8). If there is less than 20%, dentoalveolar ankylosis will not generate imaging signs yet.

When finding cementum and dentin, bone cells do not distinguish them from bone trabeculae and cortex, and continue their bone-remodeling saga to adapt to the immediate and necessary functional demands (Figs 2 to 9).

## There is no pus formation in dentoalveolar ankylosis and replacement resorption; only where there are bacteria

The inflammatory exudate in the form of pus is the result of the exuberant interaction between staphylococcal and streptococcal bacteria with neutrophil-type leukocytes^6^. These bacteria are known as pyogenic, and these leukocytes as pyocytes, because the pus-forming process is peculiar to them.

The interaction between bacteria and neutrophils releases many degradative enzymes from tissue proteins at a very acidic pH and with many toxic substances. In that environment, there are no cellular and tissue building activities of synthesis and construction; there is only destruction.

In dentoalveolar ankylosis and replacement root resorption, there are normal tissue activities, such as bone remodeling, characterized by alternate resorption and bone neoformation.^7^ The pH is neutral or tends to be predominantly basic. Thus, there is no way to exist simultaneity between bone remodeling and osseointegration in an environment where there are bacteria. In any tissue environment, the formation of pus results from the interaction of bacteria with neutrophils. There is no formation of pus without bacteria.

## Teeth just do not remodel because cementoblasts and ERM stop tem

Bone remodeling just does not involve the teeth because of the following factors:

1. The protection offered by the epithelial rests of Malassez and the released EGF, as previously described.
2. Cementoblasts coating the roots, cells of a lineage similar to that of osteoblasts but without surface receptors to interact or “listen” to the mediators that command the speed of bone remodeling.

Once these two protective structures are eliminated from dental roots, the bone will include teeth in the process of bone remodeling and will penetrate on the root structure (Fig 2), incorporating dentin and dental soft tissues - pulp and periodontal ligament - as part of its structure (Figs 3 to 9). This process following dentoalveolar ankylosis is called replacement root resorption.

As bone remodeling is constant, to the point of completely renewing the skeleton after 5 to 7 years in a young adult, an ankylosed tooth will take between 1 and 10 years to disappear completely, by the process called replacement root resorption. This happens in a context of tissue normality, without pain and without other symptoms. There will be neither pulpitis, nor increased dental mobility, nor will the tooth color change.

## Dental implants do not hamper bone remodeling

Particulate products applied in the body can be classified into three types,^6^ as to their interaction with cells and tissues:

1. Antigens or immunogens: when they have proteins in their composition, which gives them an ability to induce inflammation and immune response simultaneously, producing antibodies and sensitive cells that will seek to eliminate them. Classical examples are microorganisms and transplanted organs.
2. Foreign bodies: when they do not have proteins in their composition and, therefore, when entering the tissues they promote only inflammation, with no associated immune response. The inflammation, via macrophages, will remain around indefinitely, on its surface, trying to phagocytose and eliminate them, without giving up. At the periphery of these macrophages, clustered around this foreign body, a delicate fibrous capsule will be formed to isolate the process from the rest of the body. Foreign bodies do not induce immune responses, such as immunorejections. As an example, one may mention metals, gutta-percha cones, filling cements, suture threads, among others.
3. Inert bodies: represented by materials that, when entering tissues and contacting their cells and structures, induce neither inflammatory response nor immune response. They are considered by the tissues as a structural part of that anatomical region. Thus, they can be used to fill spaces, give volume and even to withstand anchoring and/or chewing forces.

Titanium-based dental implants are classic examples of inert bodies. Applied in areas of bone repair, cells and neoformed tissue interact with their surface and integrate them in such a way that they become part of their organizational and physical structure. In the tissue, bone remodeling process just does not reabsorb titanium dental implants and does not replace them due to our cells inability to phagocytose and process metals; but their surface fully integrates with the bone, in the phenomenon known as osseointegration.

In other words, when one has ankylosis, the tooth will be gradually replaced by bone, because dental tissues are resorbable, or “phagocytable” by the clasts after 1 to 10 years: one day the tooth will become bone and its structures will disappear.

## Dentoalveolar ankylosis and replacement resorption do not interfere with osseointegration. Why could decoronation followed by dental implants placement be a very interesting therapeutic alternative?

**Figure 10:**
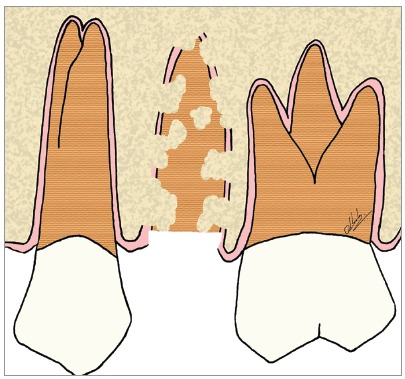
Scheme of decoronation in the maxillary second premolar, with replacement resorption, preparing it to receive an implant in the region.

**Figure 11:**
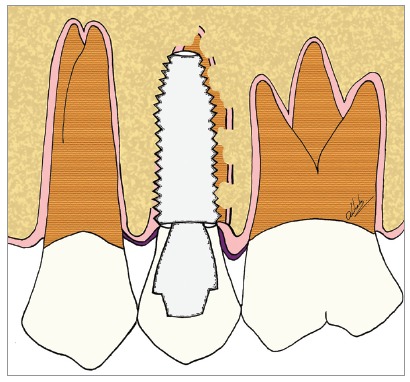
Scheme of decoronation in the maxillary second premolar, with replacement resorption, followed by the installation of an implant in the region. The replacement of dental remnants will follow its natural course and will not disrupt osseointegration.

In dentoalveolar ankylosis and replacement root resorption, once established and without contamination of the region, it can be affirmed that dental tissues can, and should, be considered as normal bone to be remodeled (Fig 2, 3 and 4). What can be expected from the bone can also be expected from the dental tissues merged with this bone. This bone with ankylosed dental tissues and undergoing bone replacement can be the place for surgical procedures - such as, for example, osseointegrated dental implants (Figs 10 and 11). The osseointegration process will occur normally and the biological show will go on. 

**Figure 12:**
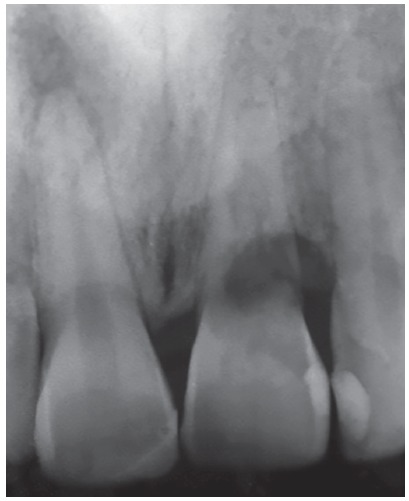
Diagnostic radiography of the maxillary left central incisor with replacement resorption induced by dental trauma, whose prognosis is the inevitable tooth loss. The right central incisor also suffered trauma, but without progression to dentoalveolar ankylosis, and evolved to aseptic pulpal necrosis.

**Figure 13:**
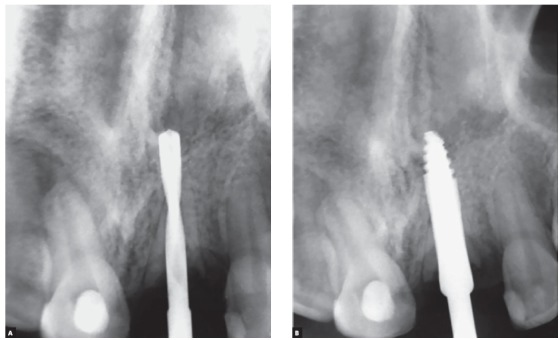
Radiographs of intermediate surgical maneuvers, preparing the place to receive the dental implant, in the patient of the previous figure.

**Figure 14:**
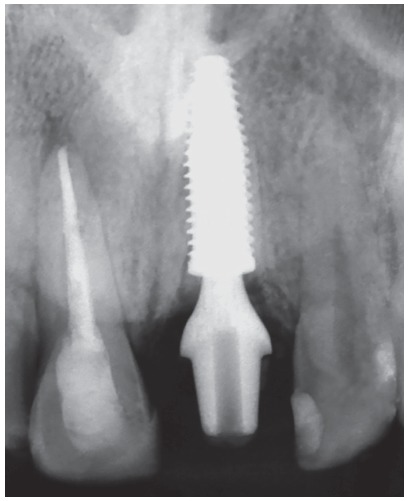
Final radiography of the site after installing the implant in the decoronated central incisor region with replacement resorption. The adjacent central incisor also suffered trauma, but without progression to dentoalveolar ankylosis, and evolved to aseptic pulp necrosis, endodontically treated.

**Figure 15:**
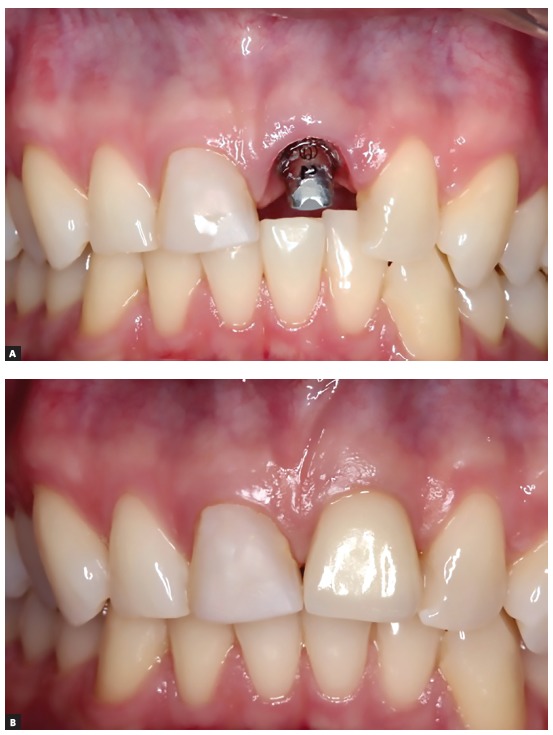
Clinical aspects before and after the fixation of the coronary part of the prosthesis on the implant installed in the decorated tooth.

**Figure 16:**
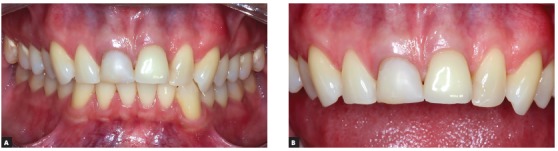
Buccal clinical aspects six years after the procedure.

**Figure 17:**
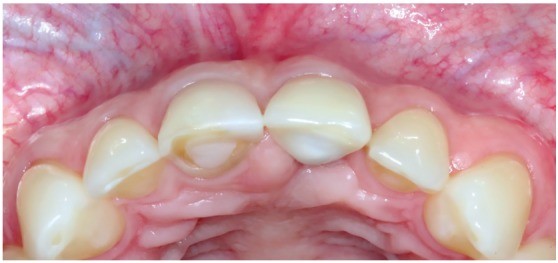
Clinical aspects, in occlusal view, six years after the procedure. It is noted the volume and the gingival contour maintenance, as well as the normal color and texture.

**Figure 18:**
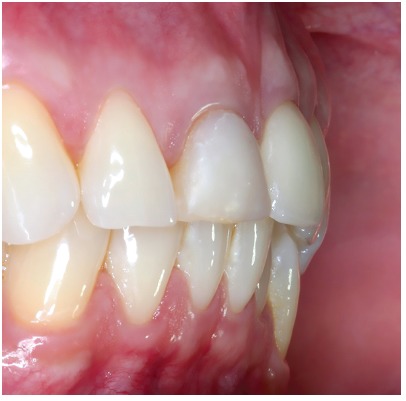
Clinical aspects, in lateral view, six years after the procedure. It is noted the volume and the gingival contour maintenance, as well as the normal color and texture.

**Figure 19:**
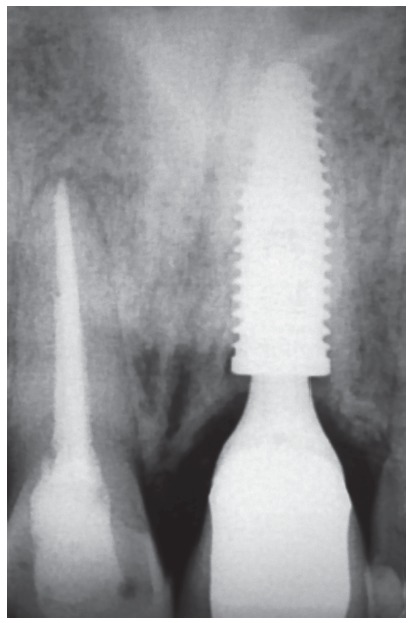
Radiographic aspects of the implant in the region of the decoronated tooth, six years after the procedure. Remnants of the tooth with replacement resorption and osseointegration are also noted.

For the installation of osseointegrated implants in the areas with dentoalveolar ankylosis and replacement resorption, it is necessary to decoronate the compromised tooth, sectioning its crown up to 2 mm below the bone level (Figs 10 to 15). Once this procedure has been performed, the cavity in which the implant will be installed should be prepared. The location, angulation and optimum positioning in the space to place the implant should be dictated exclusively by the clinical convenience associated with the previous planning. In other words, planning should be done considering the area as consisting of bone that will receive an implant, “forgetting” that there are teeth with replacement resorption - as seen in the clinical case presented in Figures 16 to 19.

It should be noted that, on the surfaces of the implants that touch dentin areas, there will be a mechanical overlap and, gradually, they will be replaced by the adjacent bone. There is no analogous process to that of osseointegration that can be called “dentin integration “, because there will be no dentin formation on the surface of the implants, since odontoblasts will not be formed in this area to the point of promoting dentinogenesis. The term “dentin integration” should not be used as it is inappropriate and conceptually unfounded.

One of the advantages of the decoronation followed by dental implants placement in teeth with ankylosis and replacement resorption is the maintenance of the bone volume in the region, both vertical and horizontal. If it is possible to preserve the buccal part of the root, even if very thin, in the cavity preparation for the implant, this will leave the gingival tissues looking completely normal for long periods.

The decoronation success regarding the occurrence of osseointegration is independent of the type and brand of the dental implant. The occurrence of dentoalveolar ankylosis and replacement resorption on the site should not change the planning for implant installation or even the criteria for choosing the type and brand of dental implant to be used. There is no report of failure after decoronation and implant placement.^17^

In order to emphasize and show how the decoronation of teeth with replacement resorption immediately followed by the installation of osseointegrated implants should be considered a valid therapeutic alternative, Figures 12 to 19 show the clinical case of a 26-year-old patient and his clinical control after 6 years of follow-up, at 32 years of age.

## How about the dental pulp? What if the tooth has previous endodontic treatment?

Loose, fibrous, cartilaginous, adipose and bony connective tissues, when operated by instruments such as scalpels and curettes, after internalized by mucosa and overlying skin or by dressings and prosthetic pieces, naturally evolve for repair, which returns their functions and needed demands.

Inflammation with pus and pain formation occurs only if there is bacterial contamination in the site, with interaction of neutrophils and bacteria, establishing a purulent exudate. When the pulp is sectioned in conservative procedures, such as pulpotomies and curettage, there is no pus formation and the repair is established in the operated pulp areas.^5^

After the decoronation and/or during the process of replacement resorption in teeth with pulp vitality (Fig. 3), the pulp and periodontal tissues are organized into fibrous connective tissue and integrate medullary spaces. Cells with odontoblasts phenotype in their proliferative processes will now differentiate into osteoblasts, fibroblasts and other cells of mesenchymal origin, such as odontoblasts and their predecessor cells. Remember: phylogenetically, dental tissues come from bone tissue, primitively.

If the tooth with dentoalveolar ankylosis and replacement resorption presents endodontic treatment and no bacterial contamination (Fig 4), the cones and filling cements should be removed at the time of decoronation. If the cavity to be determined for the implant placement vertically involves that part of the root containing the endodontic treatment, such a maneuver may become unnecessary.

It is important in the selection of cases for decoronation the absence of microbial contamination in the region, which may be represented by the presence of chronic periapical lesions, fistulas, old unconsolidated root fractures and active advanced periodontal disease.

In the first studies on decoronation^13^, it was recommended that, in teeth with live pulp, the endodontic instruments should be conducted to the end of the canal to promote bleeding in the apical tissues of the root involved. In fact, biologically, this need is not grounded, making this maneuver dispensable. The root should only be sectioned (Figs 10 to 15).

## Other indications, applications and clinical explanations

Directly related uneruptured teeth involving neural branches, such as the inferior alveolar, can be sectioned only in their crowns very close to the oral environment or to the mandibular second molar. Decoronation is also referred to as coronectomy. The sectioned root portion in an uncontaminated bone environment may remain isolated indefinitely and, if it progresses to dentoalveolar ankylosis and replacement resorption, it will disappear over the years. In this way, post-surgical sensitivity changes, which may be irreversible, can be prevented.

Removal of non-erupted teeth in very aberrant locations - such as close to the condyle, orbit, or even the mandible base - may weaken the region or promote anatomical and functional sequelae. In such cases, removing these teeth does not constitute an urgency or emergency; one can control peripheral tissues with periodically obtained images. Over time, there may be sharp atrophy of the periodontal ligament (which is usually only 0.25mm thick), causing bony bridges to dribble epithelial rests of Malassez and to install dentoalveolar ankylosis and replacement resorption.

If it is necessary for an implant to pass or reach the area where the non-erupted teeth are, one can trespass them with these implants. In general, these surgical procedures represent surgical tooth injuries that lead the affected teeth to ankylosis and replacement resorption without impeding the implants’ osseointegration and leading, over the years, to their disappearance, by replacing teeth by bone.

## FINAL CONSIDERATIONS

1. Uncontaminated maxillary areas that present teeth with dentoalveolar ankylosis and replacement root resorption can be considered as bearing normal bone, in continuous process of remodeling, and osseointegrated implants can be successfully installed. Bone remodeling will promote osseointegration, regardless of whether there are roots in ankylosis and/or replacement resorption. After 1 to 10 years, all dental tissues will be replaced by bone.

2. The location, angulation and optimal positioning in the space to place the implant should be dictated exclusively by the clinical convenience associated with previous planning. In other words, make a plan considering this area as consisting of normal bone to receive an implant; disregard, or “forget,” that there are teeth with replacement resorption.

3. One of the advantages of decoronation with the installation of implants in ankylosed teeth with replacement resorption is the maintenance of the bone volume in the region, both vertical and horizontal. When preparing the implant cavity, if possible, preserve the buccal part of the root, even if thin; this will leave gingival tissues looking quite normal for long periods.

4. The important thing in case selection for the decoronation is the absence of microbial contamination in the region, which can be represented by the presence of chronic periapical lesions, fistulas, old unconsolidated root fractures and active advanced periodontal disease. These situations are contraindications to decoronation.

5. The occurrence of dentoalveolar ankylosis and replacement root resorption without contamination should not change the planning for implant placement, nor even the criteria for choosing the type and brand of dental implant to be used. No failure in decoronation with subsequent use of dental implants has been reported.
